# Prosthetic complications of fixed dental prostheses supported by locking-taper implants: a retrospective study with a mean follow-up of 5 years

**DOI:** 10.1186/s12903-021-01843-2

**Published:** 2021-09-27

**Authors:** Wen mo Gao, Wei Geng, Chen chen Luo

**Affiliations:** Department of Dental Implant Center, Beijing Stomatological Hospital, School of Stomatology, Capital Medical University, No. 4 Tian Tan Xi Li, Dongcheng District, Beijing, 100050 People’s Republic of China

**Keywords:** Prosthetic complications, Implant restorations, Implantology, Locking-taper implants

## Abstract

**Background:**

Restoration with locking-taper implants is a widely used methodology. However, conical connection systems such as locking-taper implant systems have rarely been examined. This study provides a retrospective investigation of locking-taper fixed restorations, mainly focusing on prosthetic complications.

**Methods:**

Patients undergo treatment with conical connected implants from 2008 to 2010 were examined. Preparation of the implant sites was performed according to the standard procedures for the Bicon system. Bone healing took over 6 months, and the prosthetic procedure was initiated thereafter. Integrated abutment crowns or gold porcelain crowns were used, and the prosthesis type was a single crown or a fixed dental prosthesis. Once the crown was in place, its occlusion was thoroughly checked and adjusted, and then the crown was glazed or finely polished. The Kaplan–Meier method was used to calculate the cumulative complication-free rates for 5 and 10 years. Additionally, a Cox regression model was used to identify the factors that independently influenced the results. Implant survival and marginal bone loss were also investigated.

**Results:**

A total of 392 patients who underwent 541 implants and 434 locking taper implant-based restorations from 2008 to 2010 were examined. The overall 5-year cumulative complication-free rate was 83.34%. The most common prosthetic complication was veneer chipping, with a frequency of 67.53%. According to the Cox regression model, the complication-free rate of integrated abutment crowns was significantly higher than that of gold porcelain crowns, that of molar regions was significantly higher than that of premolar regions, and that of females was significantly higher than that of males. Only three implant failures happened, and the mean marginal bone loss values at 1- year, 5-years and 10- years were 0.25 mm (95% CI ± 0.12), 0.40 mm (95% CI ± 0.03) and 0.51 mm (95% CI ± 0.05), respectively.

**Conclusion:**

Veneer chipping was the most common complication with locking-taper implant-supported fixed restorations. The incidence of complications for IACs is significantly higher than that for GPCs. Age, location, and prosthesis type are not determinants of prosthetic complications. Besides, the long-term clinical effect of locking-taper implant can meet the clinical needs. The bone tissue level around the implant can maintain long-term stability.

## Background

Implant treatment is becoming an increasingly popular choice for patients. A study showed that the implant survival rate of implant supported fixed prostheses (single crowns) was satisfactory [1]. However, complications arising from implant treatment are a bothersome issue for both doctors and patients [1, 2]. Additionally, it has been reported that screw failure is a large concern for clinicians and patients [3]. As shown by a systematic review, 12.7% of implant supported fixed prostheses are affected by loosening after an average of 5 years [4]. Screw loosening can be caused by inadequate tightening torque, fatigue, the settling effect, micromotion, and excessive bending [5]. Thus, an appropriate preload is crucial for the joint stability. Although screw loosening does not necessarily lead to prosthesis failure, it can allow plaque deposits to form that result from microgaps and micromotion [6, 7], and these deposits can lead to further biological complications, such as peri-implantitis or peri-implant mucositis. Accordingly, pure (screwless) implant systems, such as conical connection systems that are fixed by only friction, have been developed.

The implant-abutment interface of the conical connection system is mostly Morse taper connected with cold-welding [8, 9], thus eliminating the prosthetic complications associated with screws [10]. The implant-abutment connections were usually less than 1.5° Morse tapers with an internal cone [8]. The abutment is fixed only by means of friction. Moreover, cold-welding provides a well-closed abutment-implant interface, which is conducive to plaque control and may reduce the incidence of biological complications [11]. Compared to screw-based systems, locking-taper connections are more stable and can better resist lateral and axial forces [8, 12].

Many studies and systematic reviews [13, 14] have assessed the complication rates of implant-supported fixed prostheses. However, most of these earlier studies focused on butt-joint screw- type implant-abutment connection systems, while relatively few have examined conical connection systems [15, 16]. The present study was a long-term retrospective study that aimed to assess the correlations between the cumulative prosthetic complication-free rate of fixed prostheses supported by locking-taper implants and various relevant factors, such as patient age, patient sex, prosthesis position, jaw position, restoration type (single crown (SC) or fixed partial prosthesis crown (FDP)), and the prosthetic materials that were used. The marginal bone loss (MBL) and implant survival rate were also investigated.

## Methods

### Study design and sample

The current work was a long-term retrospective clinical study with an average follow-up of 5 years. All patients who were referred to the Beijing Stomatological Hospital during 2000–2010 and received at least one Bicon (type of implant type, Bicon, Boston, MA, US) implant were enrolled in the study. This article has been reported using a statement from STROBE (Strengthening the Reporting of Observational studies in Epidemiology) as closely as possible. This study was performed in accordance with the Declaration of Helsinki and was approved by the Ethics Committee (Scientific Research/Technical Branch) of Beijing Stomatological Hospital affiliated with Capital Medical University (Approval Number: CMUSH-IRB-KJ-PJ-2019-12). Informed consent was obtained from all the participants enrolled in the present study.

The exclusion criteria were as follows:

1. Patients for whom the implant failed in regard to osseointegration before the prosthetic procedures;
2. Patients with implants supported over dentures;
3. Patients whose medical records cannot provide sufficient information (specific restoration materials);
4. Patients with CAD-CAM zirconia or non-precious metal porcelain crowns;
5. Patients for whom a guided bone regeneration (GBR) procedure was used.
6. Patients for whom a sinus floor elevation procedure was used.

The relevant factors that were considered were age > 60 years or 18 years ˂age < 60 years, sex, implant position (anterior, premolar, or molar region), implant location (maxilla or mandible), prosthesis type ( single crowns(SCs) or fixed dental prosthesis (FDP), and restoration material (integrated abutment crowns(IACs) or gold alloy porcelain crowns(GPCs)). Information was collected from the patients' medical records and disaggregated based on the relevant factors to identify those that had an impact on the incidence rate of prosthetic complications. Specific definitions of the complications are presented in Table 1. Both IACs and GPCs are most commonly used at our hospital, while non-precious metal porcelain and zirconia crowns are seldomly used. Therefore, the latter two types of crowns were not included in the study. Since the present study mainly focused on the prosthetic complications, implants that failed before prosthetic procedures started were excluded. Implants that demonsteated failed osseointegration, i.e., within 6 months of the implant operation, were excluded. To make the marginal bone loss (MBL) values more comparable, patients for whom a sinus floor elevation procedure and a guided bone regeneration (GBR) procedure were used were excluded.

**Table 1:** Definition of the complications

Prosthetic complications	Definition
Veneer chipping	Veneer chipping or the fracture of IACs or GPCs, metal base exposed or not
Abutment loosening	Loosening that occurred in implant abutment interface, not in abutment-restoration interface
Abutment fracture	Fracture on the abutments, including cracks or complete fracture
Crown decementation	Decementation in GPCs

This study focused mechanical complications, including veneer chipping/fracture, abutment loosening/fracture, and restoration decementation. Specific definitions of the complications are presented in this table

### Surgical and restorative procedures

All implants were Bicon (type of implant type, Bicon, Boston, MA, US) American implants. The implant-abutment connections were 1.5° locking tapers with an internal cone. Preparation of the implant sites was performed according to the standard procedures for the Bicon system. Bone healing took over 6 months, and the prosthetic procedure was initiated thereafter. All the impressions were taken using polyether silicone rubber (type of rubber type, 3 M, St. Paul, MN, US). IACs or GPCs were used, and the prosthesis type was an SC (single crown) or an FDP (fixed dental prosthesis). Integrated abutment crowns (IACs) are a typical and fully retrievable type of restoration that is supported by locking-taper implants. IACs are metal-resin crowns made from a titanium base and a composite resin veneer of Ceramage (type of veneer resin, SHOFU, JP). Ceramage(Type of veneer resin, SHOFU, JP) is a fiber-reinforced composite resin with filler particles that has a higher strength than conventional resin [17]. The GPCs were cemented with glass ionomer cement. Once the crown was in place, its occlusion was thoroughly checked and adjusted, and then the crown was glazed (GPCs) or finely polished (IACs).

### Clinical and radiographic examination

This study focused on prosthetic complications, including veneer chipping/fracture, abutment loosening/fracture, and restoration decementation. These complications were recorded as endpoint events. Biological complications were not examined in the present study. Implant failures were counted in this study. Implant failures in this study were defined as the failures occurring after prosthetic loading, and other failures occuring before prosthetic loading were not included. Implant failures in this study included peri-implantitis, progressive bone loss and implant body fracture.

Intraoral periapical radiographs were taken for each implant at 1-, 5- and 10- year follow-up examinations time respectively for comparative analysis and measurements (Digimizer, ver.4.3.4, MedCalc Software, Ostend, Belgium). The distance from the most coronal bone to the margin of the implant neck was measured. To eliminate the impact of the distortion of images, the distance was calculated as the ratio of the implant length measured on the radiograph to the actual implant length. Mesial and distal distances were measured for each implant, and the average of the two was considered as the final MBL. The process was repeated by the same observer for three times, and the final result was the average of the three measurements.

### Data management and statistics

A database was prepared using Microsoft Excel. The populations of the different variable groups were assessed, and the data were then transferred to GraphPad Prism (version 7.0) for statistical analysis. Kaplan–Meier estimators were used to obtain survival curves (restoration based), from which the cumulative complication-free rates were calculated (95% CI). Log-rank and Gehan-Breslow-Wilcoxon tests were used to assess whether there were significant differences in the survival curves between the different groups (p < 0.05 represented a significant difference). In addition, implant failures and MBL were also investigated.

Based on the statistical results, the factors that had an impact on prosthetic complications were identified. After single-factor analysis was performed, multifactor analysis using a Cox regression model (SPSS version 23.0) was used to identify the factors that independently influenced the results. Factors with a p value < 0.1 were included in the Cox regression model.

## Results

### Preliminary statistical results

A total of 392 patients who had at least one Bicon implant installed between January 2008 and January 2010, including a total of 541 implants and 434 restorations (201 for male patients and 233 for female patients), were included in the study. The average age of the patients was 43.07 years (restoration-based). The patient distribution is shown in Table 2.

**Table 2:** Study variables

Study variables		%
N %		
Gender		
Male	201	46.31
Female	233	53.67
Age		
Age > 60	72	16.59
18 < age < 60	362	83.41
Material		
IAC	139	32.03
MC	295	67.97
Type		
SCs	327	75.35
FDPs	107	24.65
Implant position		
Anterior region	32	7.37
Premolar region	94	21.66
Molar region	308	70.97
Implant location		
Maxilla	209	48.16
Mandible	225	51.84

The relevant factors were considered to be age (> 60 years or < 60 years), gender, implant position (anterior, premolar, or molar region), implant location (maxilla or mandible), prosthesis type (SC or FPPC), and restoration material (IAC or GPC). Distribution by different factors are shown in Table 1

**Fig. 1:**
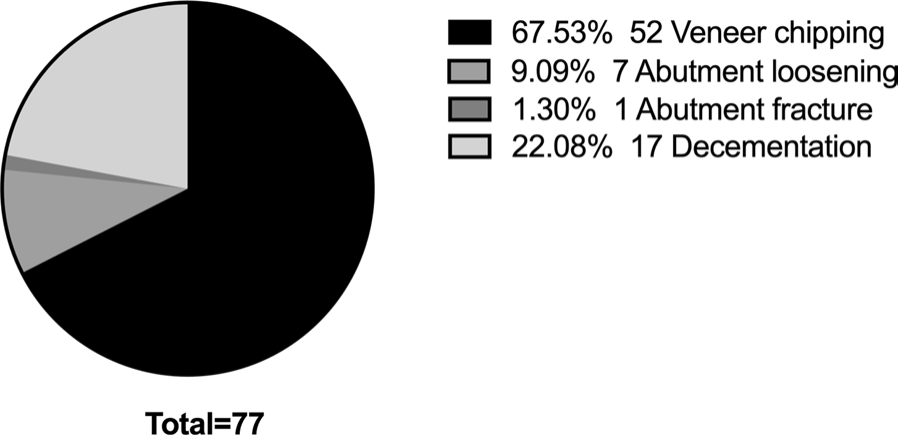
Prosthetic complications distribution. In total, 77 prostheses suffered from the prosthetic complications. The most common mechanical complication is veneer chipping (52), followed by decementation (17) (which only occurrs in GPCs). The specific complication frequencies are presented in Fig. 1

The complication distribution by time is shown in Table 3. Between 2008 and 2010, 77 of the 434 restorations were affected by prosthetic complications. Other mechanical complications, such as implant fracture, were not included since no implant fractures occurred. The most common prosthetic complication was veneer chipping (52), with a frequency of 67.53%. Only one abutment fracture occurred in the 3rd year after the prosthesis placement. The complication frequencies are presented in Fig. 1.

**Table 3:** Total prosthetic complications

Complication	≤ 2 years	2–5 years	> 5 year	Total
Veneer chipping	17	19	15	52
Abutment loosening	0	2	5	7
Abutment fracture	0	1	0	1
Crown loosening/shedding	6	8	3	17

Complication distribution by time is shown in Table 3. Totally 78 of 451 prostheses suffered from prosthetic complications. Two single IACs suffered from veneer chipping within 3 months, and these cases are counted as early restoration failures. Specific statistics are shown as above

A total of 3 implant failures occured in 3 patients, and details are shown in Table 4. The MBL values at the 1-, 5- and 10-follow up were 0.25 mm (95% CI ± 0.12 mm), 0.40 mm (95% CI ± 0.03 mm), 0.51 mm (95% CI ± 0.05 mm), respectively (Table 5).

**Table 4:** Failed implants

Gender	Age	Site	Time of failure(after prosthetic loading) (years)	Type of failure
M	56	36	2	Peri-implantitis
M	49	26	1	Peri-implantitis
F	52	47	4	Peri-implantitis

A total of 3 implant failures happened in 3 patients respectively, details were shown in Table 4. All the 3 implants failed in 5 years after loading

**Table 5:** Marginal bone loss disaggregated by time

Year	Mean	SD	Median	95% CI
1	0.25	0.25	0.24	0.13–0.37
5	0.40	0.29	0.41	0.37–0.43
10	0.51	0.30	0.52	0.46–0.56

A mean MBL at 1-year, 5-year and 10-year was 0.25 mm (95% CI ± 0.12), 0.40 mm (95% CI: ± 0.03) and 0.51 mm (95% CI ± 0.05), respectively. The bone tissue level around the implant can maintain long-term stability

### Univariate survival analysis

**Fig. 2:**
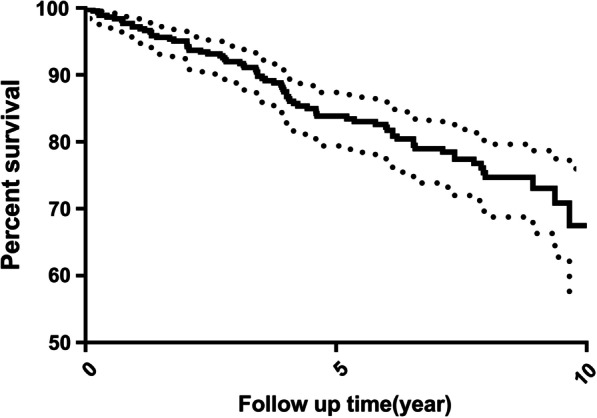
Overall prosthesis mechanical complication-free survival curve

The overall prosthesis survival curve is presented in Fig. 2. The overall 5-year cumulative complication-free rate was 83.84%, while the 10-year rate was 67.48% (Table 6). Significant differences in the survival curves were observed in the material and position groups (p < 0.05). Conversely, no significant differences were observed in the sex, age, location, or prosthesis-type groups (p > 0.05). There were significant differences in the early phase for the position groups (Gehan–Breslow–Wilcoxon test: p = 0.02). For the location groups, prostheses in the mandibles showed a slightly higher complication incidence rate than those in the maxilla (p = 0.09). Furthermore, the female group had a slightly higher complication rate than the male group (p = 0.05), and the older group had a slightly higher complication rate than the younger group (p = 0.33). However, these differences were not statistically significant. No notable differences were observed between these groups (p = 0.67). Nevertheless, these factors may influence each other. Multivariate analysis with Cox regression was used to identify the factors that independently influenced the results. To avoid the omission of possible influential factors, factors with a p value < 0.1 were included in the Cox regression model, including sex (p = 0.0526), material (p = 0.04), implant position (p = 0.04) and location (p = 0.09) (Table 6).

**Table 6:** Cumulative complication-free rates and curve comparison

Study variables	Kaplan–Meier		Log-rank test		Gehan–Breslow–Wilcoxon test	
	5y	10y	Chi-square	P-value	Chi-square	P-value
Gender						
Male	87.0%	73.0%	3.758	0.0526	3.358	0.07
Female	80.87%	64.05%				
Age						
> 60	76.23%	67.47%	0.946	0.33	1.235	0.27
< 60	85.03%	67.28				
Material						
IAC	79.49%	53.06%	4.356	0.04	3.283	0.07
GPC	85.04%	73.31%				
Type						
SCs	84.01%	54.70%	0.181	0.67	0.215	0.64
FPPs	83.53%	75.27%				
Implant position						
Anterior region	85.02%	77.94%	6.703	0.04	7.542	0.02
Premolar region	93.17%	85.51%				
Molar region	81.27%	64.22%				
Implant location						
Maxilla	87.38%	78.20%	2.936	0.09	2.073	0.15
Mandible	80.20%	62.00%				
Total	83.84%	67.48%			–	

Cumulative complication-free rates and statistical features are shown as above. The overall 5-year cumulative "complication-free" rate is 83.84%, while the 10-year rate is 67.48%. Significant differences are observed in material and position groups

### Multivariate regression analysis

Multivariate regression analysis with Cox regression was used to examine the influential factors, including sex, material, implant position and location. The results are shown in Table 7. After multivariate regression analysis, the results showed that material, gender and tooth position were independent factors affecting the survival time. According to the HR value, the material was an independent protective factor, indicating that IACs had a longer survival time than GPCs (p = 0.043, HR = 0.615 (95% CI [0.385;0.984])). Sex was an independent risk factor, and the female survival time was shorter than the male survival time (p = 0.041, HR = 1.627 (95% CI [1.021;2.592])). The survival time of premolars was longer than that of molars (p = 0.024, HR = 0.375 (95% CI [0.160;0.879])). The other indicators did not independently affect the survival time.

**Table 7:** Cox regression model of the four influencing factors

Influencing factors	*B*	SE	Wald	*P*	HR	HR 95% CI
Material	− 0.486	0.240	4.106	0.043	0.615	0.385–0.984
Location	0.184	0.244	0.565	0.452	1.201	0.745–1.939
Gender	0.487	0.238	4.192	0.041	1.627	1.021–2.592
Position						
Molar region			5.098	0.078		
Anterior region	− 0.08	0.476	0.028	0.867	0.923	0.363–2.348
Premolar region	− 0.98	0.435	5.09	0.024	0.375	0.160–0.879

Multivariate regression analysis with Cox regression was used to examine the influential factors, including gender, material, implant position and location. After multivariate regression analysis, the results showed that material, gender and tooth position were independent factors affecting survival time

## Discussion

A previous 5-year systematic review of the clinical outcomes of fixed complete dentures supported by implants showed that the survival rate of the implants was satisfactory but that the prosthetic complication rate was rather high [18]. Veneer chipping was the most common prosthetic complication. Other studies have also shown that the complication rates of implant-supported fixed prostheses have significantly increased over time [2, 19]. In most clinical studies regarding locking-taper systems, most of the prostheses that were examined were porcelain-fused-metal-fixed prosthetics, most commonly GPCs [15, 20, 21]. Compared to the number of studies on GPCs, far fewer studies have focused on metal-acrylic prostheses, and most of these studies have mainly focused on complete dentures [22, 23]. The conclusion that veneer chipping is the most common prosthetic complication associated with titanium-acrylic complete dentures has been confirmed in other studies [22–24].

In the current study, prosthetic complications, such as abutment loosening and abutment fracture, occurred much less frequently than technical complications such as veneer chipping or decementation. This prosthetic complication distribution is similar to that observed in a previous retrospective study, in which only two abutment fractures and one case of abutment loosening were observed in the 80 subjects [10].

Screw-connected implant systems can have microgaps of approximately 40–100 μm at the interface between the implant and abutment [25, 26], which will accumulate plaques and increase the probability of peri-implantitis [6]. Locking-taper implant systems can greatly reduce the microgaps (1–3 μm) compared to the former and thus may decrease the probability of peri-implantitis [11]. The implant survival in this study is satisfactory. Several other studies confirm that among all of the patients who met the inclusion criteria, only three implant failures occurred. All the three failed implants were peri-implantitis, which supported single crown prostheses. Although peri-implantitis occurred, the morbidity was greatly reduced compared to screw-connected implant systems [1, 27, 28], which may be ascribed to the minute microgap of locking-taper implant systems. The present study revealed a stable MBL, at 1 year, 5 years and 10 years. Due to the high dropouts of the present study, the 10-year MBL values are for reference only. The results of MBL in the present study are similar to those of previous studies, which indicates that the long-term clinical outcome of locking-taper implants is satisfactory [16, 29]. The results of MBL of the present study are similar to those of previous studies, which indicates that the long-term clinical outcome of locking-taper implant is satisfactory.

The results of the multifactor analysis are shown in Table 7.According to a previous study, the use of Ceramage can reduce the risk of veneer chipping in comparison to that of conventional metal-based porcelain crowns since its elastic modulus is close to that of natural teeth [30]. However, in the present study, compared with the GPCs, the survival time of the IAC crowns was significantly shorter, which was consistent with the results of the univariate analysis. The interfacial adhesion of titanium and acrylic is mainly due to physical adhesion, and some chemical adhesion is provided by coupling agents. These types of adhesion are weaker than the chemical adhesion between gold alloy and porcelain. We believe that these different interfacial bonding forces are the root cause of the results presented in this study. Similarly, a recently published meta-analysis reported that the chipping rate of porcelain-fused-metal-fixed prostheses is 9%, which is much lower than that of metal-resin prostheses (27%) [31]. However, prosthetic complications, such as abutment loosening and abutment fracture, were presented by GPCs. These results may be the result of the cushioning effect of the resin, which accommodates some of the mechanical force that is applied to implant components. IACs have unique advantages. The resin exhibits excellent stress resistance, reducing damage to the implant [32].The bonding of IACs is completed outside of the mouth, reducing the potential irritation of the periodontal tissue by the adhesive. Additionally, the fact that IACs can be repaired in the mouth by clinicians can greatly reduce procedure times. In contrast, the repair of metal-based porcelain crowns, including alternatives such as GPCs, can be difficult. The strength of the metal base can be reduced by repeated firings, during which tiny cracks may arise [33].

After removing the confounding factors, there was no significant difference in the survival time of the prosthesis in the maxilla and mandible, and the difference between the two groups was further narrowed, which may indicate that the effect of the jawbone density was not as large as expected. The molar region presented higher complication rates than that of the premolar region, and a significant difference was observed (log-rank test p < 0.05). Similarly, it has been previously reported that prosthetic complications occur more frequently in the molar region because of the mechanical force conditions in that area [34]. Since the number of cases in the anterior tooth area was too small, the results of the anterior tooth area in this study were not statistically significant. A study have showed that the incidence of complications in the anterior tooth area was slightly lower than that in the posterior tooth area [20]. This may be related to the inclusion of both biological and repair complications in the study.

In contrast to the univariate analysis, there was a significant difference in survival time between the sex groups in the multivariate analysis. This may be because females typically exhibit better adherence than females and return more quickly for advice than men when complications occur, or the result may have been affected by other influencing factors.

This study was a retrospective survival analysis, and the results will inevitably deviate from those of prospective experiments. The sample size of some groups in this study was small. For some variable groups, the differences were rather significant, such as those between the restoration type (FDPs: SCs = 107: 327), material (IACs: GPCs = 139:295), and age (> 60 years: < 60 years = 72: 362). These differences may have impacted the statistical results to a certain degree. Biological complications and the survival rate require further prospective and long-term follow-up studies.

## Conclusions

Veneer chipping is the most common prosthetic complication for both IACs and GPCs that are supported by locking-taper implants. Over time, the incidence of complications for IACs is significantly higher than that for GPCs. Significant differences were observed between the different prosthetic materials, placement positions and sexes. The bone tissue level around the locking-taper implant can maintain long-term stability.
